# Association Between hsTnT and NT-proBNP and Peripheral Artery Disease in People with HIV: A Multicentre Danish Cohort Study

**DOI:** 10.3390/biom15030401

**Published:** 2025-03-11

**Authors:** Thomas R. Holtveg, Anne Marie Reimer Jensen, Ask Bock, Moises Alberto Suarez-Zdunek, Andreas D. Knudsen, Børge G. Nordestgaard, Shoaib Afzal, Thomas Benfield, Sisse R. Ostrowski, Tor Biering-Sørensen, Ruth Frikke-Schmidt, Susanne D. Nielsen

**Affiliations:** 1 Department of Infectious Diseases, Copenhagen University Hospital—Rigshospitalet, 2100 Copenhagen, Denmark; 2 Department of Cardiology, Copenhagen University Hospital—Herlev and Gentofte Herlev, 2730 Herlev, Denmark; 3 Department of Clinical Biochemistry, Copenhagen University Hospital—Herlev and Gentofte, 2730 Herlev, Denmark; 4 Department of Clinical Medicine, Faculty of Health and Medical Sciences, University of Copenhagen, 2100 Copenhagen, Denmark; 5 Department of Infectious Diseases, Copenhagen University Hospital—Amager and Hvidovre, 2650 Hvidovre, Denmark; 6 Department of Clinical Immunology, Copenhagen University Hospital—Rigshospitalet, 2100 Copenhagen, Denmark; 7 Department of Biomedical Sciences, Faculty of Health and Medical Sciences, University of Copenhagen, 2100 Copenhagen, Denmark; 8 Department of Cardiology, Copenhagen University Hospital—Rigshospitalet, 2100 Copenhagen, Denmark; 9 Steno Diabetes Center Copenhagen, 2730 Herlev, Denmark; 10 Department of Clinical Biochemistry, Copenhagen University Hospital—Rigshospitalet, 2100 Copenhagen, Denmark; 11 Department of Surgery and Transplantation, Copenhagen University Hospital—Rigshospitalet, 2100 Copenhagen, Denmark

**Keywords:** peripheral arterial disease, NT-pro B-type natriuretic peptide, high-sensitivity troponin T, HIV, biomarker

## Abstract

People with HIV (PWH) have a high risk of peripheral artery disease (PAD), and high-sensitivity troponin (hsTnT) and NT-pro B-type natriuretic peptide (NT-proBNP) may be useful biomarkers for PAD in PWH. We assessed associations between hsTnT and NT-proBNP and both prevalent PAD and de novo PAD. Adult PWH were examined at baseline and after 2 years. Inclusion criteria were (1) measurements of hsTnT and NT-proBNP at baseline and (2) ankle brachial index (ABI) at baseline for prevalent PAD and both visits for de novo PAD. PAD was defined as ABI ≤ 0.9. We included 1011 PWH, and 88 (8.7%) had PAD at baseline. Among 802 PWH, 29 (3.6%) had de novo PAD at follow-up. A doubling in hsTnT concentration was associated with prevalent PAD with an OR 1.41 (95% CI: 1.02–1.96, *p* = 0.04) and 1.40 (95% CI: 0.99–1.98, *p* = 0.055) in a base model and an adjusted model, respectively. High hsTnT was associated with a risk ratio of 3.39 (95% CI: 1.24–9.27, *p* = 0.02) for de novo PAD in an unadjusted model and 3.44 (95% CI: 0.98–12.10, *p* = 0.05) after adjustments. NT-proBNP was not associated with PAD. Thus, hsTnT was associated with higher odds of prevalent PAD and increased risk of de novo PAD.

## 1. Introduction

People with HIV (PWH) have twice the risk of atherosclerotic cardiovascular disease compared to the general population [1]. This also includes a higher risk of peripheral artery disease (PAD) [2]. PAD is a manifestation of atherosclerosis that is characterised by compromised blood flow to the legs. This may result in symptoms such as muscle aches, leg weakness, ulcerations, and may ultimately lead to amputation [3]. Early diagnosis of PAD is essential to improve the prognosis of PAD [4].

The most common method to diagnose PAD is the ankle-brachial index (ABI) [5]. While ABI measurements are a sensitive and time-efficient method, ABI measurements are not routinely performed in primary care settings [6,7]. A biomarker that identifies PWH at high risk of PAD could be used to screen and decide which patients to refer for an ABI measurement in a specialised centre.

The cardiac isoform of troponin T is a protein that leaks into the blood stream in the event of tissue damage from cardiac muscle cells and skeletal myocytes [8]. This is not the case for troponin I, since it is only expressed in cardiac muscle [9]. High-sensitivity troponin T assays (hsTnT) are commonly used in risk stratification, as a diagnostic tool and in prognostic models for cardiovascular disease [10,11]. B-type natriuretic peptide (BNP) is a hormone secreted in the event of cardiac wall stress. BNP can counteract the renin-angiotensin-aldosterone system and limit cardiac fibrosis [12,13]. High N-terminal pro B-type natriuretic peptide (NT-proBNP) is associated with increased mortality [14], and NT-proBNP can be used in the diagnosis and prognosis of heart failure [15]. Studies from general population cohorts have shown associations between high hsTnT and NT-proBNP and a higher risk of PAD [16,17,18]. Previous studies have found that PWH have higher concentrations of both hsTnT and NT-proBNP than controls [19,20], but the association between these biomarkers and PAD among PWH has not been explored.

The aim of this study was to determine whether high concentrations of hsTnT and/or NT-proBNP were associated with prevalent PAD in PWH. Furthermore, we aimed to assess whether high concentrations of these biomarkers were associated with increased risk of de novo PAD among PWH. We hypothesised that high concentrations of both hsTnT and NT-proBNP were associated with prevalent PAD and increased risk of de novo PAD.

## 2. Materials and Methods

### 2.1. Study Design

This study is based on data from the Copenhagen Comorbidity in HIV infection study (COCOMO). COCOMO is an observational longitudinal cohort study of PWH [21]. Participants were examined at two timepoints: first at baseline (March 2015–November 2016) and then at a 2-year follow-up visit (April 2017–April 2019). For this study, we included all COCOMO participants with an ABI measurement, hsTnT and NT-proBNP measured at baseline. Participants with an ABI ≥ 1.4 were excluded, as this may indicate arterial stiffening and limits the accuracy of ABI as a diagnostic tool [5]. For analyses of de novo PAD, only participants without prevalent PAD at baseline and measurement of ABI at the 2-year follow up visit were included.

All participants provided informed consent both orally and in writing. The ethics committee of the Capital Region of Denmark (H-8-2014-0004) and the Danish Data Protection Agency (30–1454) approved the COCOMO study.

### 2.2. Biochemical Analysis

Plasma samples (EDTA-anti-coagulated) were collected from venous blood at baseline and stored at −80 °C until measurement of hsTnT and NT-proBNP (2023). hsTnT and NT-proBNP analyses were carried out in plasma using electrochemiluminescence-immunoassay on Roche Cobas 8000, e801 module with the Elecsys Troponin T hs kit and the Elecsys proBNP II kit (Roche, Rotekreuz, Switzerland) in Rigshospitalet Copenhagen. The CVmax was 7% at 20 ng/L and 5% at 23 pmol/L (194.5 ng/L) for hsTnT and NT-proBNP analysis, respectively. Interleukin-6 (IL-6) was analysed with a multiplex assay (Meso Scale Discovery, Rockville, MD, USA) in suitable EDTA-plasma 2 years after storage at −80°. HbA1c, plasma glucose, triglyceride and hsCRP were analysed in plasma immediately after collection on Cobas 8000 (Roche) at Copenhagen University-Herlev and Gentofte, Herlev, Denmark. Low-density lipoprotein cholesterol (LDL) was calculated using the Friedewald equation when triglycerides in plasma were below 4.0 mmol/L [22] and otherwise measured directly.

### 2.3. Blood Pressure Measurements

The blood pressure was measured twice on each arm after 15 min of rest using an automated sphygmomanometer (IntelliValue MP5SC, Phillips, Amsterdam, The Netherlands) with the highest systolic blood pressure (SBP) used. Ankle SBP was measured in the supine position using a Doppler device (Sonotrax Basic A 294534; San Diego, CA, USA) and a manual sphygmomanometer (DuraShock Silver Ds-6501-300; Welch Allyn, Skaneateles Falls, New York, NY, USA) in either the posterior tibial artery or the dorsalis pedis artery. Ankle SBP was measured twice, and the highest measurement was used. ABI was calculated separately for each leg as a ratio of ankle SBP to arm SBP. The lowest ABI of the two legs was used in the statistical analysis in accordance with the European Society of Cardiology (ESC) guidelines [5].

### 2.4. Other Covariates

Demographic variables, use of antihypertensives, antidiabetics and smoking habits were obtained from self-reported questionnaires. Unfilled questions in the questionnaire were regarded as “No” when applicable. Body mass index (BMI) was calculated using height and weight [23] measured by trained medical staff at the baseline visit. HIV-specific variables including ART usage, viral load, date of HIV diagnosis and CD4+ T cell counts (CD4 count) and information on history of CVD were obtained through review of medical records by trained personnel. History of CVD was defined as a previous diagnosis of atrial fibrillation, acute or chronic coronary syndrome, heart failure with both preserved and reduced ejection fraction, valvular disease, stroke and/or coronary artery bypass graft surgery.

### 2.5. Definitions

hsTnT was categorised into three groups: low (≤6 ng/L), medium (>6 and ≤14 ng/L) and high (>14 ng/L). A threshold of 14 ng/L corresponds to the 99th percentile of a healthy population [24] and aligns with the intervals used in similar studies [16,17]. NT-proBNP was divided into two groups: low (≤15 pmol/L (126.9 ng/L)) and high (>15 pmol/L (126.9 ng/L)) in accordance with ESC guidelines [15].

Prevalent PAD at baseline was defined as ABI ≤ 0.9. De novo PAD was defined as ABI > 0.9 at the baseline visit and ≤0.9 at the 2-year follow-up visit. Hypertension was defined as SBP ≥ 140 mmHg and/or diastolic blood pressure ≥ 90 mmHg and/or use of antihypertensive drugs at baseline. Diabetes was defined as HbA1c ≥ 48 mmol/mol or a non-fasting glucose ≥ 11.1 mmol/L or use of anti-diabetic drugs at baseline. BMI was divided into categories following the WHO guidelines [23]: <18.5 kg/m^2^ = underweight, 18.5–24.9 kg/m^2^ = normal weight, 25–29.9 kg/m^2^ = overweight and ≥30 kg/m^2^ = obese.

### 2.6. Bias

Our study design could have introduced selection bias as only PWH who were followed at a hospital were invited to join the cohort. Therefore, PWH with poor adherence were less likely to attend the cohort study and our data are not generalisable to this group.

### 2.7. Statistical Analyses

The normality of the data was assessed visually. Log transformation was used when model assumptions were not met. Normally distributed continuous data were presented as means and standard deviations (SD). Non-normally distributed continuous data were presented with medians and interquartile ranges (IQR). Categorical data were reported as counts and percentages. Data were censored from the tables if the characteristics had fewer than 5 participants to secure anonymisation. Between-group comparisons for continuous data were performed with the Student’s *t*-test or the Mann–Whitney U test where appropriate. Categorical data were compared using the χ2-test.

Logistic regression models were used to assess the association between hsTnT or NT-proBNP and PAD at baseline and presented as odds ratios (ORs). hsTnT and NT-proBNP were assessed one at a time as both continuous and categorical explanatory variables in two models: (1) a base model adjusted for age and sex, and (2) a model adjusting for age, sex, smoking, hypertension and diabetes. These variables were chosen a priori.

The association between biomarkers and de novo PAD at the 2-year follow-up visit was assessed with Poisson regression with robust standard errors and presented as risk ratios (RRs). Again, hsTnT and NT-proBNP were assessed one at a time as both continuous and categorical explanatory variables in two models: (1) a completely unadjusted model and (2) an adjusted model with adjustment for age, smoking and diabetes. The low number of de novo PAD cases only allowed for minimal adjustment due to risk of overfitting and the a priori model could not be used. We therefore employed a stepwise forward selection using the variables in the cross-sectional analysis to find the adjusted model for the analysis of de novo PAD. To further validate the robustness of our findings, we performed sensitivity analyses by excluding participants with incomplete smoking data or history of CVD and by sequentially adding total cholesterol, HDL, education, alcohol intake and physical activity to the model assessing the association between hsTnT, prevalent PAD and de novo PAD.

Analyses were performed using R (version 4.3.2) with the ggplot2 package. A two-sided *p*-value below or equal to 0.05 was regarded as statistically significant.

## 3. Results

### 3.1. Baseline Characteristics

**Figure 1:**
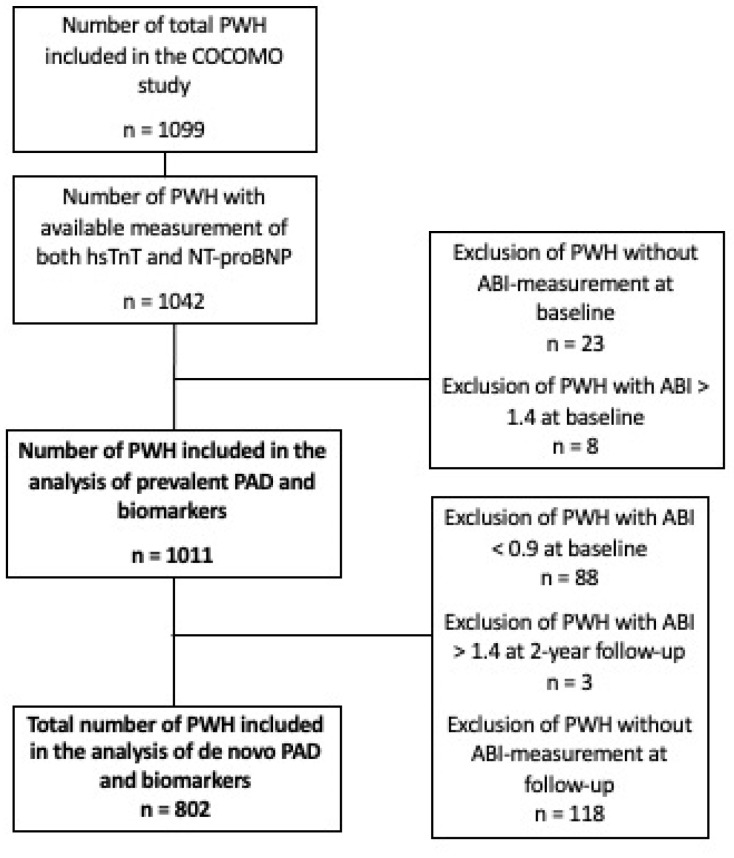
Flowchart of the study inclusion from the COCOMO cohort. The COCOMO cohort included a total of 1099 PWH of whom 1011 had available measurement of hsTnT and NT-proBNP. PWH with an ABI above 1.4 were excluded. For the analysis of de novo PAD, PWH with PAD at baseline were excluded as well as PWH who did not have an ABI at the 2-year follow-up. Finally, PWH with an ABI above 1.4 at the 2-year follow-up were excluded, which resulted in a total of 802 PWH. Abbrevations: ABI = Ankel Brachial Index, PAD = Peripheral artery disease, PWH = People with HIV.

The cross-sectional analysis of prevalent PAD included 1011 PWH from the COCOMO study, and 802 were included in analysis of de novo PAD (Figure 1).

As reported previously, at baseline, 88 (8.7%) had prevalent PAD, and 29 (3.6%) developed de novo PAD during the 2-year follow-up visit [24,25]. PWH with prevalent PAD were older, had lived with HIV for a longer duration, were more likely to be current smokers and had higher prevalence of hypertension compared with PWH without PAD at baseline (Table 1).

Median hsTnT at the baseline visit was 5.32 ng/L, and 61 (6%) had high hsTnT. PWH with high hsTnT were older, predominantly male, had lived with HIV for a longer duration, had higher prevalence of hypertension and diabetes and had a lower current CD4 count than PWH with low hsTnT (Table 2). Median NT-proBNP at baseline was 5.18 pmol/L (43.81 ng/L), and 111 (11%) had high NT-proBNP. PWH with high NT-proBNP were older, predominantly male, had lived with HIV for a longer duration, had a higher prevalence of hypertension and had a lower current CD4 count than those with low NT-proBNP. However, those with high NT-proBNP had a lower prevalence of diabetes (Table 2).

Participants with prevalent PAD, high hsTnT and high NT-proBNP were older, had higher prevalence of hypertension, more were smokers and their concentration of IL-6 was higher (Table 1 and Table 2). Participants with prevalent PAD had a lower median hsTnT compared to the median of the categorized group of high hsTnT (Table 1 and Table 2).

### 3.2. Prevalent PAD

**Figure 2:**
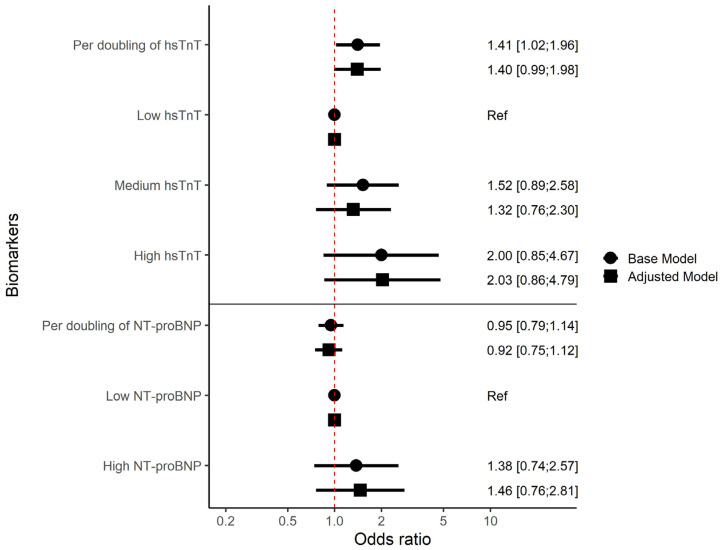
Cross-sectional associations between hsTnT, NT-proBNP and prevalent PAD among 1011 PWH at baseline. Results are presented as odds ratios. hsTnT and NT-proBNP are presented as both continuous and categorical variables. Continuous variables are shown as per doubling of concentration and categorical variables are shown compared to “low”. The numbers show the estimated odds ratio with following confidence intervals in brackets. Base model: adjusted for age per 10 years and sex, adjusted model = adjusted for age per 10 years, sex, smoking, hypertension and diabetes. Abbreviations: hsTnT = high-sensitivity troponin T, NT-proBNP = N-terminal pro B-type natriuretic peptide, Ref = Reference category.

Among PWH, a doubling in hsTnT concentration was associated with 41% higher odds of having prevalent PAD (odds ratio (OR) 1.41 [95% confidence interval (CI): 1.02–1.96], *p* = 0.04). In the adjusted model, the adjusted OR (aOR) was 1.40 [95% CI: 0.99–1.98 with *p* = 0.05 (Figure 2). As a categorical variable, medium hsTnT was not associated with higher odds of PAD compared to low hsTnT (Figure 2). We found some evidence to suggest PWH with high hsTnT to have higher odds of prevalent PAD compared to individuals with low hsTnT (OR 2.00 [95% (CI): 0.85–4.67], *p* = 0.11) and (adjusted OR 2.03 [95% CI: 0.86:4.79], *p* = 0.11) but neither the base nor the adjusted model were statistically significant. No association was found between NT-proBNP and prevalent PAD (Figure 2).

In sensitivity analyses limited to participants without history of CVD, results were consistent (Supplemental Table S1). Sensitivity analyses with further adjustments for total cholesterol, HDL, education, alcohol intake and physical activity in both stepwise and fully adjusted model did not significantly alter our results (Supplemental Table S3). Exclusion of participants who did not respond to the questionnaire about smoking showed consistent results (Supplemental Table S5).

### 3.3. De Novo PAD

Participants with de novo PAD at the 2-year follow-up were more likely to have CD4 nadir < 200 and had higher concentrations of hsCRP and IL-6 compared to those who did not develop PAD (Table 3).

**Figure 3:**
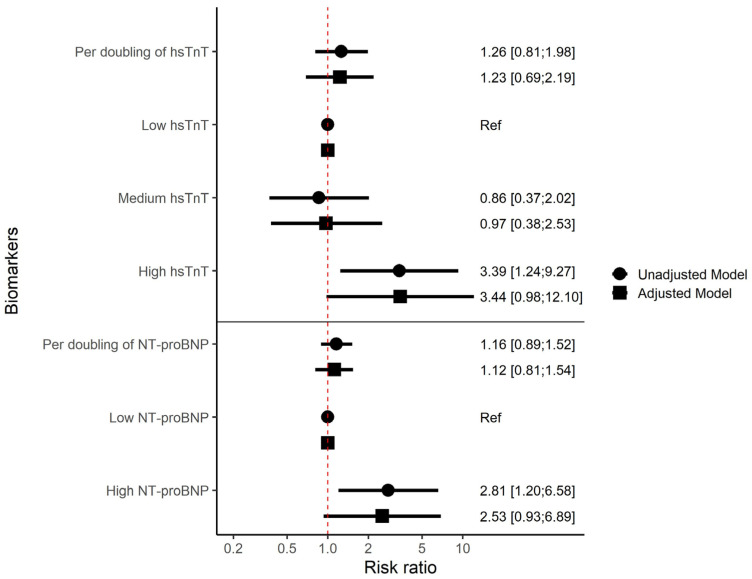
Association between hsTnT, NT-proBNP and de novo PAD among 802 PWH at 2-year follow-up. Results are presented as risk ratios. hsTnT and NT-proBNP are presented as both continuous and categorical variables. Continuous variables are shown as per doubling of concentration and categorical variables are shown compared to “low”. The numbers show the estimated risk ratio with following confidence intervals in brackets. Unadjusted = no adjustments. Adjusted model = adjusting for age per 10 years, smoking and diabetes. Abbreviations: hsTnT = high-sensitivity troponin T, NT-proBNP = N-terminal pro B-type natriuretic peptide, Ref = Reference category.

Doubling in hsTnT concentration was not associated with an increased risk of de novo PAD at the 2-year follow-up visit when modelled as a continuous variable (Figure 3). When modelled as a categorical variable, PWH with high hsTnT had a 3.39 95% CI: 1.24–9.27, *p* = 0.02 higher risk of de novo PAD compared to PWH with low hsTnT. In the adjusted model, the adjusted RR (aRR) was 3.44 [95% CI: 0.98–12.10], *p* = 0.055. Medium hsTnT was not associated with de novo PAD compared to low (Figure 3).

NT pro-BNP as a continuous variable was not associated with de novo PAD. When modelled as a categorical variable, PWH with high NT-proBNP had 2.8 times higher risk of de novo PAD (RR 2.81 [95% CI: 1.20–6.58], *p* = 0.02) compared to PWH with low NT-proBNP. In the adjusted model, the risk ratio was attenuated and was no longer statistically significant (aRR 2.53 [95% CI: 0.93–6.89], *p* = 0.07) (Figure 3). In sensitivity analyses limited to participants without history of CVD, the results were consistent (Supplemental Table S2). Sensitivity analyses with further adjustment for sex, smoking, diabetes, HDL, educational level, alcohol intake and physical activity in both stepwise (Supplemental Table S3) and fully adjusted model (Supplemental Table S4) did not significantly alter our results. Exclusion of participants who did not respond to the questionnaire about smoking showed consistent results (Supplemental Table S6).

## 4. Discussion

In this prospective cohort study, hsTnT was associated with both prevalent PAD and de novo PAD in PWH. High NT-proBNP was associated with de novo PAD in unadjusted models, although this did not reach significance in adjusted models.

We found hsTnT to be associated with both prevalent and de novo PAD in PWH. This finding could be substantial as it indicates that hsTnT has the potential to screen PWH for PAD, especially in settings where ABI measurements are not routinely performed. However, a previous study from the general population did not find an association between high hsTnT and prevalent PAD among 6016 healthy participants above 40 years of age [17]. This difference may be attributed to that fact that we also included PWH with a history of CVD, while the general population study only included participants without known CVD. Low ABI is associated with systemic atherosclerosis including coronary artery disease [25], which in turn may lead to ischemia and consequent elevation of hsTnT [26]. Thus, the observed association between hsTnT and PAD in our study may, in part, be explained by participants with other CVD such as coronary artery disease concomitant with PAD. However, a sensitivity analysis with exclusion of PWH with history of CVD, did not alter the results. This may be explained by PWH with history of CVD receive preventive treatment and that reduces their risk of de novo PAD. Another possible explanation could be due to cross-reactivity with skeletal troponin T in cardiac troponin T assays when hsTnT concentrations increase [27]. This cross-reactivity raises the possibility that the observed association between hsTnT and prevalent PAD in PWH may be a result of skeletal troponin T leakage because of compromised blood flow to the legs. Troponin I has not shown the same cross-reactivity with skeletal muscle [9] and a troponin I measurement could perhaps differentiate between tissue damage in skeletal and cardiac muscle. Unfortunately, we did not measure troponin I in our plasma samples.

Future studies should investigate the pathophysiology behind hsTnT and PAD. Despite the mechanism, PWH with high hsTnT seem to have an increased risk of PAD, and referral to an ABI measurement should be considered.

We found an association between high hsTnT and a higher risk of de novo PAD in PWH. Similarly, general population studies have found an association between high hsTnT and clinical PAD, defined as a hospital diagnosis of PAD [16,18]. One of the general population cohorts also measured ABI at follow-up visits but found no association between hsTnT and subclinical PAD (ABI below 0.9) [18]. While our study had shorter follow-up and fewer cases of PAD, we found a high risk of PAD (ABI below 0.9) associated with high hsTnT among PWH compared to the risk observed in the general population studies [16,18]. Therefore, hsTnT measurements as a screening tool for PAD could be more beneficial among PWH compared to the general population. This difference may in part be attributable to a higher inflammatory burden among PWH than the general population even when virally suppressed [28]. The higher inflammatory burden increases the risk of PAD because inflammatory markers hsCRP and IL-6 were associated with de novo PAD in another study from the COCOMO cohort [29]. These findings suggest that special attention should be made to PWH with high inflammatory markers and high hsTnT.

We found that high NT-proBNP was associated with an increased risk of de novo PAD in the unadjusted analysis, but not after adjustment for confounders. This contrasted with studies from the general population, where high NT-proBNP was associated with PAD [16,18,30]. NT-proBNP is released in response to myocardial wall stretch [31] and is associated with vulnerable plaque components [32]. Given our short follow-up, it is possible that the included PWH with PAD had not developed vulnerable plaque severe enough to influence NT-proBNP and cause PAD. The observed association is hence removed after adjustments for traditional confounders such as hypertension since NT-proBNP-elevation could be due to cardiac remodelling from a long life with traditional risk factors of CVD. Furthermore, it has been shown that PWH exhibit high markers of endothelial dysfunction [33], which indicate damage in the microvasculature resulting in high hsTnT but not NT-proBNP. This theory aligns with a study conducted in patients with type 2 diabetes where hsTnT, but not NT-proBNP, was associated with decreased capillary permeability in the peripheral microvasculature [34]. This suggests that while NT-proBNP is a well-recognised biomarker for other cardiovascular disease, hsTnT could have more potential as a biomarker for PAD risk stratification in PWH. The association found between hsTnT and PAD is significant in a clinical perspective as it could improve risk stratification among PWH for PAD prevention. However, the association could be greatly influenced by confounders. We conducted a sensitivity analysis with inclusion of total cholesterol, HDL, education, alcohol intake, and physical activity, which did not significantly alter the results (Supplemental Tables S3 and S4). However, we had few cases and results should be interpreted cautiously.

### Strengths and Limitations

The results should be interpreted considering the limitations of our study. First, the short follow-up resulted in few cases of de novo PAD at the 2-year follow-up. This limited our ability to further adjust for potential confounders and increased the risk of residual confounding. Secondly, our selection bias limits the generalisability to PWH with poor adherence. Finally, our study did not include controls so we cannot conclude if there is any difference in the association between hsTnT and NT-proBNP and PAD among PWH and healthy controls.

Biochemical analyses of NT-proBNP and hsTnT were performed on long-term stored plasma samples, which could alter the measured concentrations, and the results should be interpreted with these limitations in mind. However, long-term stability of proteins and amino acids has been demonstrated for plasma stored at −80 °C [35] and concentrations of NT-proBNP and hsTnT have both previously been shown to be stable after extended storage [36,37].

The strengths of our study include the large sample size with ABI measurements at each visit within a well-described cohort. All examinations were conducted under protocolled conditions, which secured the same proceedings at every visit. To our knowledge, this is the first study to examine the association between hsTnT and NT-proBNP and PAD in PWH.

## 5. Conclusions

In conclusion we found an association between a doubling of hsTnT and prevalent PAD in PWH. Furthermore, we found an association between high hsTnT and a higher risk of de novo PAD in PWH. NT-proBNP was associated with de novo PAD in unadjusted analysis, but not in adjusted analysis. Thus, hsTnT may have potential as a risk stratification for PAD in PWH. However, additional studies with longer follow-up are needed to further investigate the clinical utility of hsTnT and NT-proBNP in PWH.

## Figures and Tables

**Table 1:** Baseline characteristics according to PAD-status.

Variable	No PAD at Baseline (n = 923)	PAD at Baseline (n = 88)	*p*-Value
Age (years), mean ± SD	49.9 ± 10.9	55.2 ± 13	<0.001
Male, n (%)	787 (85)	75 (85)	0.99
Ethnicity			
*Caucasian, n (%)*	792 (88)	76 (91)	0.55
*Other, n (%)*	112 (12)	8 (10)	
Smoking			0.013
*Never, n (%)*	346 (38)	22 (25)	
*Current, n (%)*	260 (28)	37 (42)	
*Previous, n (%)*	317 (34)	29 (33)	
Hypertension, n (%)	360 (42)	52 (61)	0.001
Diabetes, n (%)	38 (4)	<5	0.99
BMI (kg/m^2^), mean ± SD	25 ± 3.9	24.7 ± 3.8	0.48
*Underweight, n (%)*	23 (3)	<5	0.33
*Normal, n (%)*	493 (54)	46 (52)	
*Overweight, n (%)*	313 (34)	35 (40)	
*Obese, n (%)*	90 (10)	<5	
History of CVD, n (%)	35 (4)	8 (9)	0.04
Current CD4 count (cells/µL), mean ± SD	716 ± 288	740 ± 252	0.45
CD4 nadir < 200 (cells/µL), n (%)	351 (39)	44 (51)	0.056
Viral load ≥ 50 (copies/mL), n (%)	<5	<5	0.99
Time with HIV (years), mean ± SD	14.1 ± 9	18 ± 9.5	<0.001
Use of ART, n (%)	910 (99)	86 (98)	0.86
NT-proBNP (pmol/L), median [IQR]	5.2 [2.8, 8.8]	5.3 [3, 12]	0.26
NT-proBNP (ng/L), median [IQR]	44.0 [23.7, 74.4]	(44.8 [25.4, 101.5])	0.26
hsTnT (ng/L), median [IQR]	5.2 [3.8, 7.9]	7.1 [4.7, 10.5]	<0.001
LDL (mmol/L), mean ± SD	2.8 ± 1	2.7 ± 0.9	0.45
hsCRP (mg/L), median [IQR]	1.1 [0.6, 2.4]	1.5 [0.6, 2.7]	0.20
IL-6 (pg/mL), median [IQR]	1.4 [0.9, 2.2]	1.6 [1.1, 3.4]	0.03

Abbreviations: ART = antiretroviral therapy, BMI = body mass index, CVD = cardiovascular disease, hsCRP = high-sensitivity C-reactive protein, hsTnT = high-sensitivity troponin T, IL-6 = interleukin 6, IQR = interquartile range, LDL = low-density lipoprotein, NT-proBNP = N-terminal pro B-type natriuretic peptide, SD = standard deviation.

**Table 2:** Baseline characteristics by categories of hsTnT and NT-proBNP.

Variable	High-Sensitivity Troponin				NT-proBNP		
	Low (n = 591)	Medium (n = 359)	High (n= 61)	*p*-Value	Low (n = 900)	High (n = 111)	*p*-Value
Age (Years), mean ± SD	45.9 ± 9	55.6 ± 10.4	62.7 ± 12.5	<0.001	49.1 ± 10.6	60.6 ± 10.9	<0.001
Male, n (%)	474 (80)	328 (91)	60 (98)	<0.001	777 (86)	85 (77)	0.009
Ethnicity							
*Caucasian, n(%)*	485 (84)	325 (93)	58 (100)	<0.001	762 (87)	106 (97)	0.003
*Other, n(%)*	96 (17)	24 (7)	0 (0)		117 (13)	<5	
Smoking				0.04			
*Never, n(%)*	223 (38)	126 (35)	19 (31)		330 (37)	38 (34)	0.43
*Current, n(%)*	188 (32)	94 (26)	15 (25)		268 (30)	29 (26)	
*Previous, n(%)*	180 (31)	139 (39)	27 (44)		302 (34)	44 (40)	
Hypertension, n (%)	168 (31)	206 (60)	38 (62)	<0.001	354 (42)	58 (59)	0.001
Diabetes, n (%)	9 (2)	25 (7)	8 (13)	<0.001	34 (4)	8 (8)	0.13
BMI, (kg/m^2^), mean ± SD	24.8 ± 3.8	25.4 ± 4.1	25.2 ± 4.5	0.07	25.1 ± 3.9	23.8 ± 4	0.01
*Normal, n(%)*	344 (58)	168 (47)	27 (45)	0.014	469 (52)	70 (64)	0.01
*Underweight, n(%)*	12 (2)	12 (3)	<5		20 (2)	6 (6)	
*Overweight, n(%)*	189 (32)	135 (38)	24 (40)		320 (36)	28 (26)	
*Obese, n(%)*	44 (8)	43 (12)	7 (12)		88 (10)	6 (6)	
History of CVD, n (%)	<5	22 (6)	16 (26)	<0.001	24 (3)	19 (17)	<0.001
Current CD4-count (cells/µL), mean ± SD	735 ± 282	700 ± 292	652 ± 262	0.03	720 ± 280	703 ± 322	0.55
CD4 nadir < 200 (cells/µL), n (%)	184 (32)	179 (51)	32 (53)	<0.001	331 (38)	64 (58)	0.001
Viral load ≥ 50 (copies/mL), n (%)	30 (5)	17 (5.0)	6 (10)	0.26	50 (6)	<5	0.3
Time with HIV (years), mean ± SD	12.2 ± 8.2	17.1 ± 9.5	19.8 ± 8.3	<0.001	13.9 ± 8.9	18.7 ± 9.3	<0.001
Use of ART, n (%)	581 (98)	355 (99)	60 (98)	0.77	886 (98)	110 (99)	0.9
Prevalent PAD, n (%)	36 (6)	41 (11)	11 (18)	<0.001	71 (8)	17 (15)	0.015
NT-proBNP, (pmol/L), median [IQR]	4.3 [2.5, 6.8]	6.4 [3.4, 11.1]	9.5 [5.7, 19.1]	<0.001	4.5 [2.7, 7.1]	22.6 [18.6, 38.2]	<0.001
NT-proBNP, (ng/L), median [IQR]	36.4 [21.2, 57.6]	54.2 [28.7, 93.9]	80.4 [48.2, 161.7]		38.1 [22.9, 60.1]	191.3 [157.4, 323.3]	
hsTnT, (ng/L), median [IQR]	4 [3.2, 4.9]	8.3 [6.9, 10.2]	18.5 [16.1, 22.0]	<0.001	5.2 [3.7, 7.5]	9 [5.5, 12.9]	<0.001
LDL, (mmol/L), mean ± SD	2.8 ± 0.9	2.8 ± 1	2.5 ± 1	0.03	2.8 ± 1	2.5 ± 0.8	<0.001
hsCRP, (mg/L), median [IQR]	1.1 [0.5, 2.2]	1.3 [0.7, 2.6]	1.7 [1.1, 3.0]	<0.001	1.1 [0.6, 2.3]	1.9 [1.0, 5.3]	<0.001
IL-6, (pg/mL), median [IQR]	1.3 [0.9, 1.9]	1.6 [1.1, 2.6]	2.4 [1.8, 3.9]	<0.001	1.4 [0.9, 2.1]	2.1 [1.4, 3.5]	<0.001

Abbreviations: ART = anti-retroviral therapy, BMI = body mass index, CVD = cardiovascular disease, hsCRP = high-sensitivity C-reactive protein, hsTnT = high-sensitivity troponin T, IL-6 = interleukin 6, IQR = interquartile range, PAD = peripheral artery disease, LDL = low-density lipoprotein, NT-proBNP = N-terminal pro B-type natriuretic peptide, SD = standard deviation.

**Table 3:** Baseline characteristics stratified by PAD status at the 2-year follow-up.

Variable	No PAD at Follow-Up (n = 773)	PAD at Follow-Up (n = 29)	*p*-Value
Age (years), mean ± SD	50.2 (10.9)	52.9 (11.2)	0.20
Male, n (%)	663 (85.8)	25 (86.2)	0.99
Ethnicity			
*Caucasian, n (%)*	668 (88.2)	24 (88.9)	0.99
*Other, n (%)*	89 (11.8)	3 (11.1)	
Smoking			
*Never, n (%)*	291 (37.6)	9 (31.0)	0.18
*Current, n (%)*	201 (26.0)	12 (41.4)	
*Previous, n (%)*	281 (36.4)	8 (27.6)	
Hypertension, n (%)	315 (43.2)	12 (48.0)	0.79
Diabetes, n (%)	28 (3.7)	3 (11.5)	0.14
BMI (kg/m^2^), mean ± SD	25.1 (4)	24.5 (4.1)	0.48
*Underweight, n (%)*	19 (2.5)	1 (3.4)	0.98
*Normal, n (%)*	411 (53.4)	16 (55.2)	
*Overweight, n (%)*	261 (33.9)	9 (31.0)	
*Obese, n (%)*	79 (10.3)	3 (10.3)	
History of CVD, n (%)	30 (4)	<5	0.21
Current CD4 count (cells/µL), mean ± SD	708.1 (280.5)	720.7 (363)	0.81
CD4 nadir < 200 (cells/µL), n (%)	291 (38.3)	15 (53.6)	0.03
Viral load ≥ 50 (copies/mL), n (%)	35 (4.6)	1 (3.4)	0.99
Time with HIV (years), mean ± SD	14.1 (9)	16.2 (8.9)	0.23
Use of ART, n (%)	773 (100)	29 (100)	1.00
NT-proBNP (pmol/L), median [IQR]	5.1 [2.9, 8.7]	6.8 [ 2.4, 13.0]	0.50
NT-proBNP (ng/L), median [IQR]	43.2 [24.5, 73.4]	57.6 [20.3, 110.0]	0.50
hsTnT (ng/L), median [IQR]	5.3 [3.8, 7.9]	5.5 [3.8, 9.0]	0.36
LDL (mmol/L), mean ± SD	2.8 (0.9)	2.5 (0.9)	0.07
hsCRP (mg/L), median [IQR]	1.1 [0.6, 2.4]	1.7 [1.0, 3.6]	0.02
IL-6 (pg/mL), median [IQR]	1.4 [0.9, 2.1]	1.8 [1.4, 2.5]	0.04

Abbreviations: ART = anti-retroviral therapy, BMI = body mass index, CVD = cardiovascular disease, hsCRP = high-sensitivity C-reactive protein, hsTnT = high-sensitivity troponin T, IL-6 = interleukin 6, IQR = interquartile range, LDL = low-density lipoprotein, NT-proBNP = N-terminal pro B-type natriuretic peptide, SD = standard deviation.

## Data Availability

Data are available upon request to the corresponding author.

## Supplementary Materials

The following supporting information can be downloaded at: https://www.mdpi.com/article/10.3390/biom15030401/s1, Table S1. Association between hsTnT, NT-proBNP and prevalent PAD among 968 PWH without history of CVD. Table S2. Association between hsTnT, NT-proBNP and de novo PAD among 769 PWH without history of CVD. Table S3. Stepwise adjustments for the association between hsTnT and NT-proBNP with prevalent and de novo PAD. Table S4. Association between hsTnT, NT-proBNP and de novo PAD among 802 PWH when adjusting for age, smoking, diabetes, HDL, education, alcohol intake, physical activity. Table S5. Association between hsTnT, NT-proBNP and prevalent PAD among 981 PWH, with removal of participants who did not respond to smoking behavior questions. Table S6. Association between hsTnT, NT-proBNP and de novo PAD among 772 PWH, with removal of participants who did not respond to smoking behavior questions.
