# Supplementary material for: Association Between hsTnT and NT-proBNP and Peripheral Artery Disease in People with HIV: A Multicentre Danish Cohort Study
Source: Biomolecules. 2025 Mar 11;15(3):401. doi: 10.3390/biom15030401 (PMC11940728; doi:10.3390/biom15030401)
Supplement: Supplementary file 1 [file biomolecules-15-00401-s001.zip › biomolecules-3510707-supplementary.pdf]

Table S1. Association between hsTnT, NT-proBNP and prevalent PAD among 968 PWH without history of CVD.

Variable	Base Model<br>OR 95%CI, *p*-Value	Adjusted Model<br>OR 95%CI, *p*-Value
**Troponin hsTnT**		
Per doubling of hsTnT	1.37 [0.96; 1.94] *p*= 0.08	1.35 0.94; 1.95] *p* = 0.11
- Low	1	1
- Medium	1.52 [0.89;2.60] *p* = 0.13	1.34 [0.76; 2.34] *p* = 0.31
- High	1.50 [0.55; 4.08] *p* = 0.43	1.57 [0.57;4.30] *p* = 0.38
**NT-proBNP**		
Per doubling of NT-proBNP	0.94 [0.77; 1.14] *p*= 0.50	0.91 [0.73; 1.13] *p*= 0.38
- Low	1	1
- High	1.40 [0.71; 2.76] *p* = 0.34	1.52 [0.73; 3.16] *p* = 0.26

Base model adjusted for age per 10 years and sex, adjusted model was adjusted for age per 10 years, sex, smoking, hypertension and diabetes.

Table S2. Association between hsTnT, NT-proBNP and de novo PAD among 769 PWH without history of CVD.

Variable	Unadjusted Model<br>RR 95%CI, *p*-Value	Adjusted Model<br>RR 95%CI, *p*-Value
**Troponin**		
Per doubling of hsTnT	1.32 [0.81; 2.17] *p*= 0.27	1.40 [0.77; 2.54] *p* = 0.27
- Low	1	1
- Medium	0.85 [0.35;2.09] *p* = 0.72	0.97 [0.78; 1.80] *p* = 0.95
- High	3.74 [1.24; 11.28] *p* = 0.019	3.96 [1.07;14.63] *p* = 0.039
**NT-proBNP**		
Per doubling of NT-proBNP	1.21 [0.90; 1.61] *p*= 0.20	1.17 [0.82; 1.65] *p*= 0.39
- Low	1	1
- High	3.12 [1.25; 7.77] *p* = 0.015	2.64 [0.89; 7.83] *p* = 0.08

Unadjusted model had no adjustments. Adjusted model was adjusted for age per 10 years, smoking and diabetes.

Table S3. Stepwise adjustments for the association between hsTnT and NT-proBNP with prevalent and de novo PAD.

Added Variable	Association Between Prevalent PAD and Troponin After Added Adjustments Compared to Low<br>OR 95% CI, *p*-Value	Association Between De Novo PAD and Troponin After Added Adjustments Compared to Low<br>RR 95% CI, *p*-Value
	Low: 1	Low: 1
Total Cholesterol	Medium: 1.26 [0.71;2.25], *p*-value = 0.43	Medium: 1.20 [0.44;3.24], *p*-value = 0.72
	High: 1.93 [0.80;4.67], *p*-value = 0.14	High: 3.59 [0.98;13.16], *p*-value = 0.0534
	Low: 1	Low: 1
HDL	Medium: 1.27 [0.72;2.26], *p*-value = 0.41	Medium: 1.22 [0.45;3.29], *p*-value = 0.69
	High: 2.02 [0.84;4.84], *p*-value = 0.12	High: 4.10 [1.14;14.75], *p*-value = 0.031
**Education**		
- Short	Low: 1	Low: 1
- Vocational	Medium: 1.26 [0.72;2.22], *p*-value = 0.41	Medium: 0.87 [0.32;2.35], *p*-value = 0.78
- Medium length	High: 2.14 [0.90;5.09], *p*-value = 0.09	High: 4.05 [1.15;14.29], *p*-value = 0.0295
- University degree		
	Low: 1	Low: 1

Alcohol intake per week in grams	Medium: 1.23 [0.70;2.16], *p*-value = 0.48<br>High: 1.90 [0.80;4.55], *p*-value = 0.15	Medium: 1.09 [0.40;2.94], *p*-value = 0.87<br>High: 2.97 [0.72;12.18], *p*-value = 0.13
Physical activity in spare time<br>- Inactive<br>- Slightly active<br>- Moderately active<br>- Very active<br>- Unknown	Low: 1<br>Medium: 1.30 [0.74;2.28], *p*-value = 0.35<br>High: 1.90 [0.79;4.56], *p*-value = 0.15	Low: 1<br>Medium: 0.99 [0.38;2.58], *p*-value = 0.98<br>High: 3.50 [0.98;12.42], *p*-value = 0.053
All abovementioned variables in the same model	Low: 1<br>Medium: 1.10 [0.59;2.03], *p*-value = 0.76<br>High: 1.95 [0.78;4.91] 0.16	Low: 1<br>Medium: 1.59 [0.51;4.97] *p*= 0.43<br>High: 4.38 [0.90;21.36] *p*= 0.07

Odds ratios and risk ratios for prevalent and de novo PAD, respectively, for each added variable to the adjusted model with already included age, sex, smoking, hypertension and diabetes.

Table S4. Association between hsTnT, NT-proBNP and de novo PAD among 802 PWH when adjusting for age, smoking, diabetes, HDL, education, alcohol intake, physical activity.

Variable	Adjusting for Age per 10 Years, Smoking and Diabetes RR 95% CI, *p*-Value	Adjusting for Age per 10 Years, Sex, Smoking, Diabetes, HDL, Education, Alcohol Intake, Physical Activity RR 95%CI, *p*-Value
**hsTnT**		
Per doubling of hsTnT	1.23 [0.69;2.19] *p* = 0.47	1.30 [0.64;2.64] *p* = 0.46
- Low	1	1
- Medium	0.97 [0.38;2.53] *p* = 0.96	1.59 [0.51;4.97] *p*= 0.43
- High	3.44 [0.98;12.10] *p* = 0.055	4.38 [0.90;21.36] *p*= 0.07
**NT-proBNP**		
Per doubling of NT-proBNP	1.12 [0.81;1.54] *p* = 0.50	0.96 [0.64;1.45] *p* = 0.85
- Low	1	1
- High	2.53 [0.93;6.89] *p* = 0.07	1.92 [0.54;6.87] *p*= 0.32

Table S5. Association between hsTnT, NT-proBNP and prevalent PAD among 981 PWH, with removal of participants who did not respond to smoking behavior questions.

Variable	Old Results OR 95% CI, *p*-Value	New Results with Exclusion of Participants Who Did Not Respond to Smoking Behavior Questions **OR 95% CI, *p*-Value**
**hsTnT**		
Per doubling of hsTnT	1.40 [0.99; 1.98] 0.055	1.40 [0.99; 1.98] 0.055
- Low	1	1
- Medium	1.33 [0.76; 2.31] 0.32	1.32 [0.76; 2.30] 0.32
- High	2.03 [0.86; 4.79] 0.11	2.02 [0.85; 4.78] 0.11
**NT-proBNP**		
Per doubling of NT-proBNP	0.92 [0.75; 1.12] 0.41	0.92 [0.75; 1.12] 0.41
- Low	1	1
- High	1.46 [0.76; 2.81] 0.26	1.46 [0.76; 2.80] 0.26

Table S6. Association between hsTnT, NT-proBNP and de novo PAD among 772 PWH, with removal of participants who did not respond to smoking behavior questions.

Variable	Old Results<br>RR 95% CI, *p*-Value	New Results with Exclusion of Participants Who Did Not Respond to Smoking Behavior Questions<br>RR 95% CI, *p*-Value
**hsTnT**		
Per doubling of hsTnT	1.23 [0.69; 2.19] 0.47	1.23 [0.69; 2.19] 0.47
- Low	1	1
- Medium	0.97 [0.38; 2.53] 0.96	0.97 [0.37; 2.53] 0.96
- High	3.44 [0.98; 12.10] 0.055	3.43 [0.97; 12.08] 0.055
**NT-proBNP**		
Per doubling of NT-proBNP	1.12 [0.81; 1.54] 0.50	1.12 [0.81; 1.54] 0.50
- Low	1	1
- High	2.53 [0.93; 6.89] 0.07	2.52 [0.93; 6.89] 0.07
